# has_circ_CCNB1 and has_circ_0009024 function as potential biomarkers for the diagnosis of type 2 diabetes mellitus

**DOI:** 10.1002/jcla.23439

**Published:** 2020-07-06

**Authors:** Xiangfei Chen, Jingjing Yin, Fang Zhang, Tiantian Xiao, Ming Zhao

**Affiliations:** ^1^ Department of Endocrinology School of Medicine Geriatric Research Center Jinling Hospital Nanjing University Nanjing China

**Keywords:** biomarker, circular RNAs (circRNAs), diagnosis, type 2 diabetes mellitus (T2DM)

## Abstract

Circular RNAs (circRNAs) have been reported to be associated with various diseases, including type 2 diabetes mellitus (T2DM). The aim of present study was to investigate the clinical value of has_circ_CCNB1 and has_circ_0009024 in T2DM. Serum samples from patients with T2DM (n = 166) and healthy volunteers (n = 166) were recruited. Then, real‐time quantitative reverse transcription‐polymerase chain reaction (RT‐qPCR) analysis and enzyme‐linked immunosorbent (ELISA) assays were conducted to detect the expression levels of circRNAs and inflammatory factors. Furthermore, the correlation analysis and receiver operating characteristic (ROC) curve were used to evaluate diagnostic accuracy. From the results, circ_CCNB1 was significantly increased while circ_0009024 was decreased in serum samples from T2DM patients. Moreover, has_circ_CCNB1 was positively correlated with glucose (GLU), glycosylated hemoglobin (GHb), interleukin 6 (IL‐6), and tumor necrosis factor‐α (TNF‐α) while has_circ_0009024 was negatively correlated with them. Importantly, the AUC of has_circ_CCNB1 and has_and circ_0009024 was 0.9255 (95% CI = 0.8909‐0.9601) and 0.9592 (95% CI = 0.9381‐0.9803), while the AUC of combinative curve is 0.8875 (95% CI = 0.8204‐0.8547). In a word, has_circ_CCNB1 and has_circ_0009024 may exhibit as potential biomarkers for the diagnosis of T2DM.

## Graphical abstract

Circular RNAs (circRNAs) have been reported to be associated with various diseases, including type 2 diabetes mellitus (T2DM). The aim of present study was to investigate the clinical value of has_circ_CCNB1 and has_circ_0009024 in T2DM. In the present study, circ_CCNB1 was significantly increased while circ_0009024 was decreased in serum samples from T2DM patients. Moreover, has_circ_CCNB1 was positively correlated with glucose (GLU), glycosylated hemoglobin (GHb), interleukin 6 (IL‐6), and tumor necrosis factor‐α (TNF‐α) while has_circ_0009024 was negatively correlated with them. Importantly, the AUC of has_circ_CCNB1 has_and circ_0009024 were 0.9255 (95% CI = 0.8909~0.9601) and 0.9592 (95% CI = 0.9381~0.9803), while the AUC of combinative curve is 0.8875 (95% CI = 0.8204~0.8547). In a word, has_circ_CCNB1 and has_circ_0009024 may exhibit as potential biomarkers for the diagnosis of T2DM.

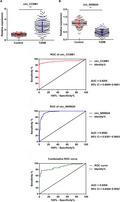

## 1 INTRODUCTION

Type 2 diabetes mellitus (T2DM), a complicated chronic metabolic disorder, is defined by insulin resistance, chronic inflammation, and hyperglycemia.^1^, ^2^ Low‐grade inflammation may result in insulin resistance, impaired glucose tolerance and finally diabetes. Meanwhile, hyperglycemia could produce and increase the expressions of inflammatory cytokines which could aggravate the insulin resistance as well.^3^ Type 2 diabetes mellitus presented a rapid increase in China and the prevalence rate of adult T2DM reached 11.6% in 2010, which was expected to increase annually.^4^, ^5^ In the advanced stage, T2DM patients often suffer from various complications.^6^ Therefore, it is urging to discover specific, sensitive, and convenient biomarkers for detecting T2DM.

Circular RNAs (circRNAs) were considered to function as the sponges of specific miRNA, regulating cellular processes at the post‐transcription stage.^7^ Accumulating evidence proved that circRNAs were associated with diabetes. For instance, has_circRNA_0054633, has_circRNA_ 063981, has_circRNA_102682, and has_circRNA_103410 were dysregulated in gestational diabetes mellitus.^8^ Moreover, 489 circRNAs were found to be dysregulated in T2DM patients and has_circRNA_00054633 presented a significant diagnostic value for pre‐diabetes and T2DM.^9^

In the present study, we focused on elucidating the clinical values of two circRNAs (has_circRNA_CCNB1 and has_circ_0009024) in detecting T2DM patients.

## 2 MATERIALS AND METHODS

### 2.1 Sample collection

In the present study, a total of 166 T2DM patients and 166 healthy volunteers were recruited from Geriatric Research Center, Jinling Hospital, Nanjing University, School of Medicine between January 2015 and December 2017 (Table 1). 2 mL blood was collected following overnight fasting and preserved in EDTA tubes. The exclusion criteria: anyone who has systematic acute or chronic inflammatory disease; cancer; renal failure; and other endocrine diseases. The inclusion criteria for T2DM were consistent with World Health Organization criteria: fasting glucose >7 mmol/L or 2 hour oral glucose tolerance test >11.1 mmol/L. Our study was approved by the Ethics Committee of Geriatric Research Center, Jinling Hospital, Nanjing University, School of Medicine, and written consent was obtained from each participant.

**Table 1:** Clinicopathological features in patients with T2DM and healthy controls

Features	Control (n = 166)	T2DM (n = 166)	*P*
Age	55.9 ± 5.6	56.4 ± 7.1	.4767
Male	96	89	1.4393
Female	70	77	
GLU (mmol/L)	5.32 ± 0.96	10.86 ± 1.55	<.0001
GHb (mg/dl)	5.51 ± 1.25	9.21 ± 1.42	<.0001

Abbreviations: Control, healthy volunteers; T2DM, type 2 diabetes mellitus; GLU, glucose; GHb, glycosylated hemoglobin.

## 3 RNA EXTRACTION AND REAL‐TIME QUANTITATIVE REVERSE TRANSCRIPTION‐POLYMERASE CHAIN REACTION (RT‐QPCR)

Total RNA was extracted from serum samples using TRIzol reagent (Thermo Fisher Scientific, Inc, USA) and reverse transcribed into cDNA under the manufacturer's instructions. Then, cDNA was amplified using the Reverse Transcription System (Promega Corporation, Madison, WI, USA) in accordance with the protocol of SYBR Green Master Mix (Takara Biotechnology Co., Ltd). The PCR thermal cycles were as follows: 35 cycles of 95℃ for 5 seconds, 62℃ for 15 seconds, and 72℃ for 20 seconds. The levels of circRNAs were normalized to U6 and analyzed using the 2^−△△Ct^ method.

## 4 ENZYME‐LINKED IMMUNOSORBENT (ELISA) ASSAY

All the serum samples were taken from the refrigerator and placed to room temperature prior to usage. The levels of IL‐6 and tumor necrosis factor‐α (TNF‐α) were measured by a Total Antigen Assay ELISA kit (Cloud‐Clone Corporation, Wuhan, China) following the manufacturer's instructions. Measurements were carried out at 450 nm.

### 4.1 Statistical analysis

All the statistical analysis was performed using the SPSS 19.0 software (IBM Corp, New York, NY) and GraphPad Prism 6.0 (GraphPad Software, Inc, La Jolla, CA, USA). Data were presented as mean ± standard deviation (SD). Significant differences between control and T2DM groups were analyzed using Student's t test followed by Newman‐Keuls post hoc test. Pearson's correlation analysis and receiver operating characteristics (ROC) analysis were used, as appropriate. *P* < .05 was considered to indicate statistically significant differences.

## 5 RESULTS

### 5.1 The expressions of has_circ_CCNB1 and has_circ_0009024 verified by RT‐qPCR

**Figure 1:**
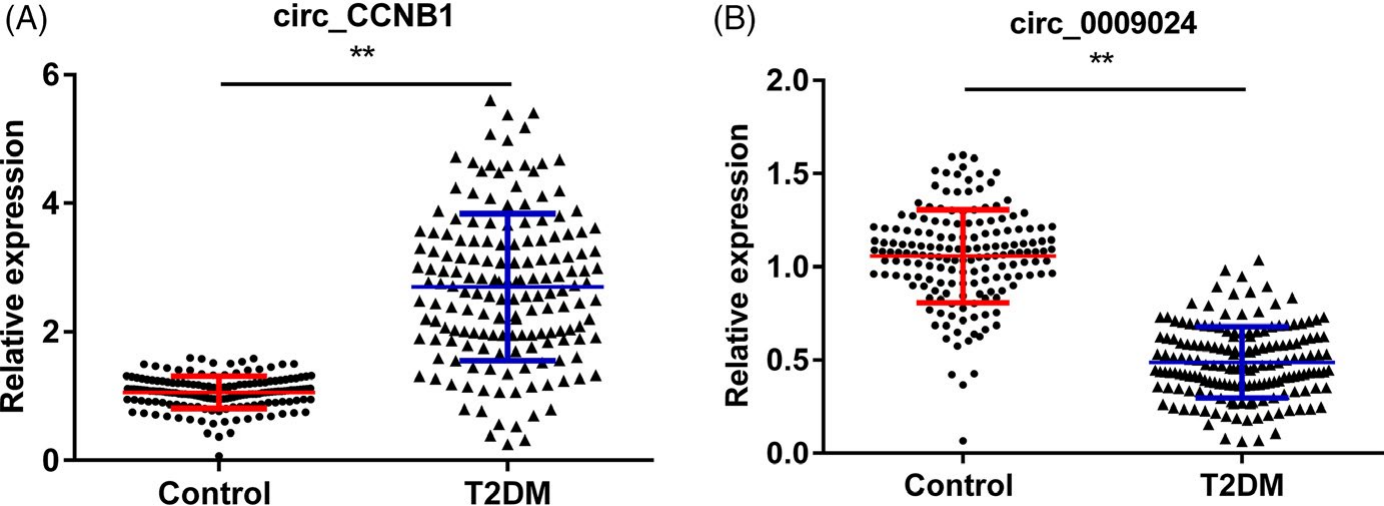
Expressions levels of has_circ_CCNB1 and has_circ_0009024. A, The expression levels of has_circ_CCNB1 in the control and T2DM groups. B, The expression levels of has_circ_0009024 in the control and T2DM groups. ^**^ *P* < .01, T2DM vs control group. T2DM, type 2 diabetes mellitus; control, healthy volunteers

To determine the expressions of has_circ_CCNB1 and has_circ_0009024 in serum samples from T2DM and healthy controls, RT‐qPCR analysis was performed accordingly. From the results (Figure 1A and B), the expression of has_circ_CCNB1 was significantly up‐regulated while has_circ_0009024 was decreased in the T2DM group compared with healthy controls (*P* < .01).

## 6 THE ASSOCIATION BETWEEN CIRCRNAS EXPRESSIONS AND GLU AND GHB

**Figure 2:**
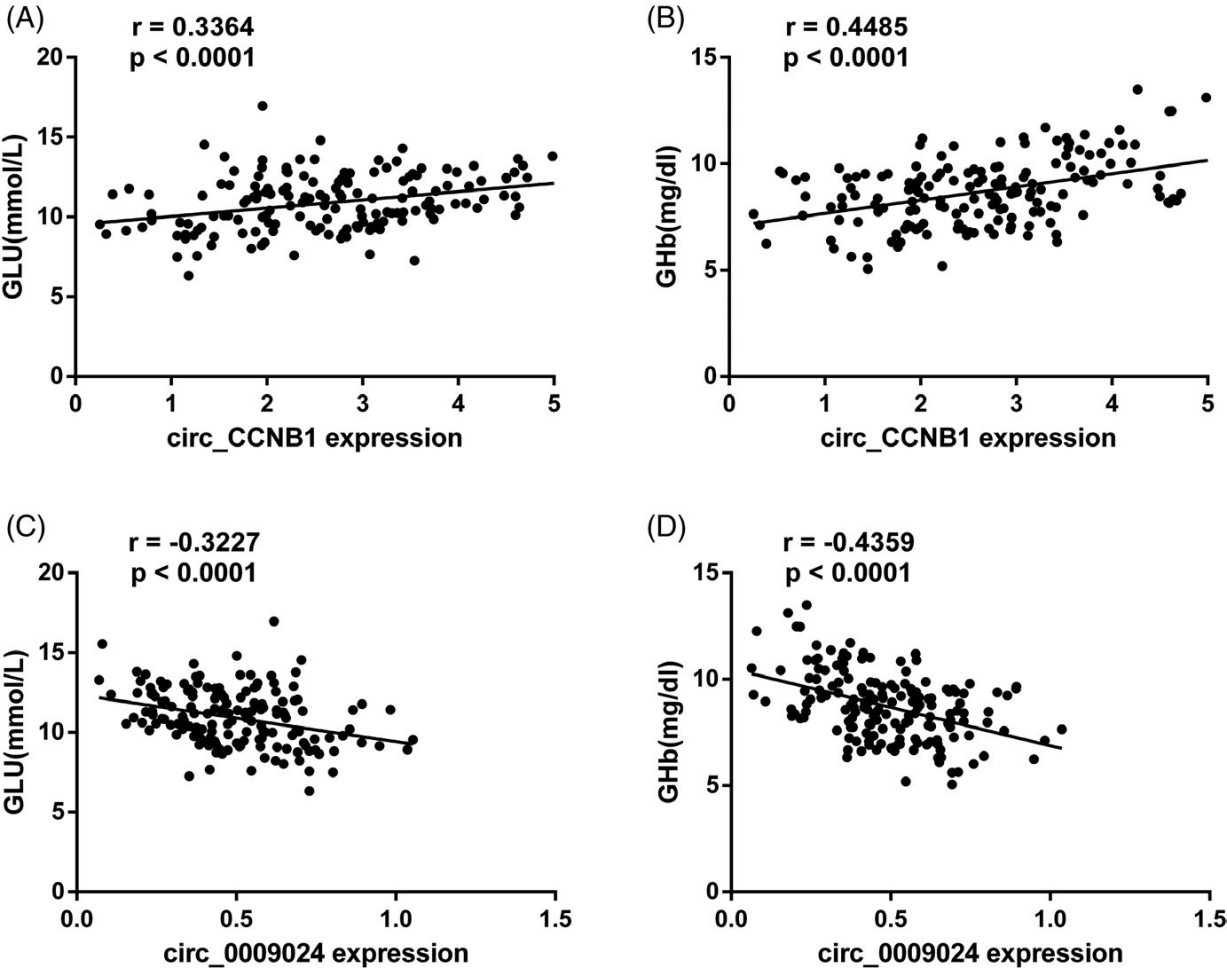
Correlation analysis between circRNAs and GLU and GHb. A, Pearson's coefficient correlation between has_circ_CCNB1 and GLU. B, Pearson's coefficient correlation between has_circ_CCNB1 and GHb. C, Pearson's coefficient correlation between has_circ_0009024 and GLU. D, Pearson's coefficient correlation between has_circ_0009024 and GHb. GLU, glucose; GHb, glycosylated hemoglobin

As shown in Table 1 and Figure 2, has_circ_CCNB1 was positively correlated with GLU (*r* = .3364, *P* < .0001) and GHb (*r* = .4485, *P* < .0001) while has_circ_0009024 was negatively correlated with GLU (*r* = −.3227, *P* < .0001) and GHb (*r* = −.4359, *P* < .0001).

## 7 THE ASSOCIATION BETWEEN CIRCRNAS EXPRESSIONS AND INFLAMMATORY FACTORS

**Figure 3:**
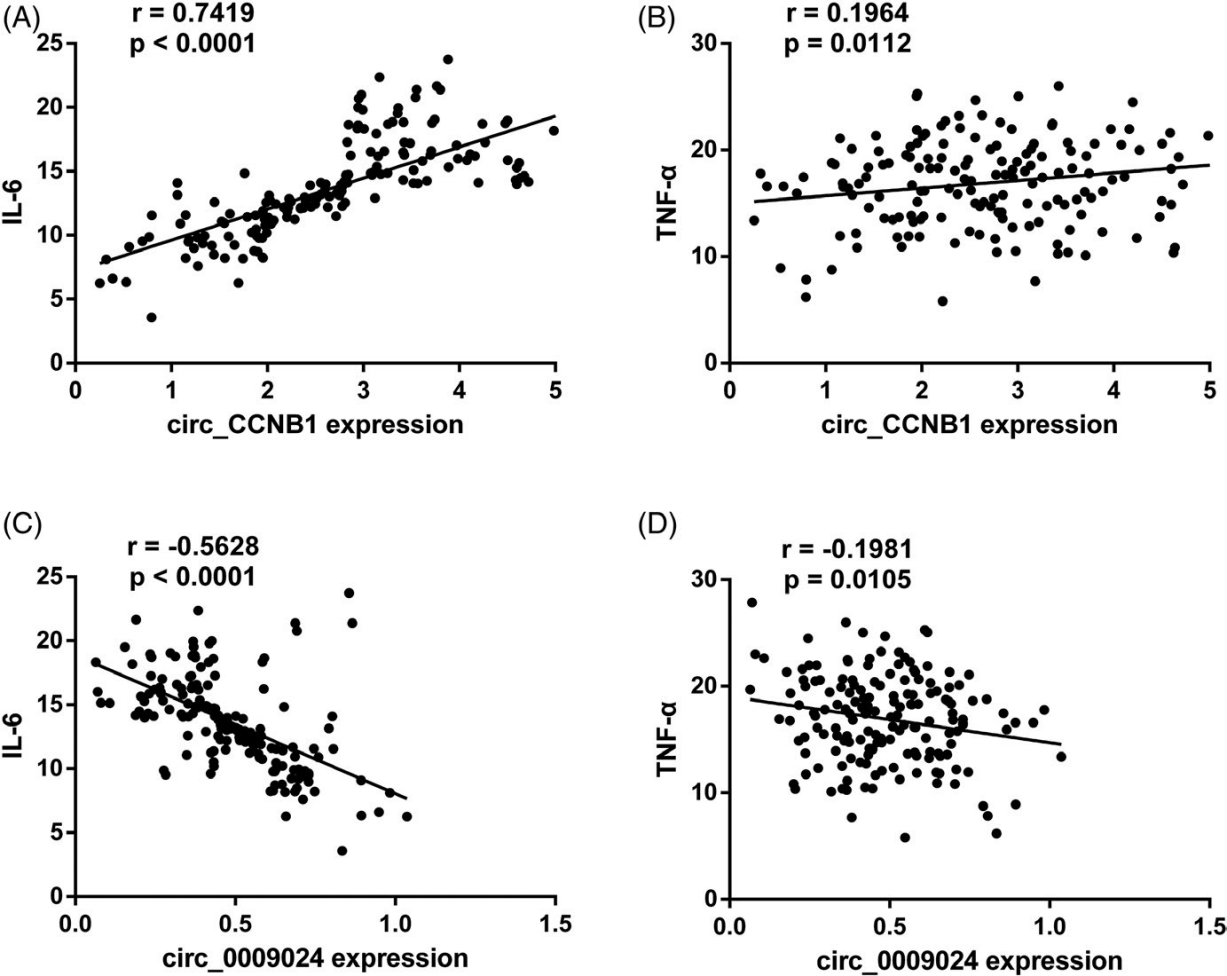
Correlation analysis between circRNAs and inflammatory factors. A, Pearson's coefficient correlation between has_circ_CCNB1 and IL‐6. B, Pearson's coefficient correlation between has_circ_CCNB1 and TNF‐α. C, Pearson's coefficient correlation between has_circ_0009024 and IL‐6. D, Pearson's coefficient correlation between has_circ_0009024 and TNF‐α. IL‐6, interleukin 6; TNF‐α, tumor necrosis factor‐α

The expression levels of inflammatory factors (IL‐6, TNF‐α) were measured by ELISA. As shown in Table 2, the expression levels of IL‐6 and TNF‐α were markedly elevated in patients with T2DM as compared with controls (*P* < .01). Furthermore, Pearson's correlation analysis was conducted to examine the correlation between circRNAs and inflammatory factors. As shown in Figure 3, has_circ_CCNB1 was positively correlated with IL‐6 and TNF‐α (*r* = .7419, *P* < .0001; *r* = .1964, *P* = .0112 respectively). However, has_circ_0009024 was negatively correlated with IL‐6 and TNF‐α (*r* = −.5628, *P* < .0001; *r* = −.1981, *P* = .0105, respectively).

**Table 2:** Expressions of inflammatory cytokines in T2DM patients and healthy controls

Features	Control	T2DM	P
IL‐6	6.25 ± 2.32	13.57 ± 3.86	<.0001
TNF‐α	11.24 ± 2.96	16.85 ± 3.91	<.0001

Abbreviations: T2DM, type 2 diabetes mellitus; IL‐6, interleukin 6; TNF‐α, tumor necrosis factor‐α.

## 8 ROC CURVE ANALYSIS OF has_circ_CCNB1 AND has_circ_0009024 WITH DIFFERENT EXPRESSIONS

**Figure 4:**
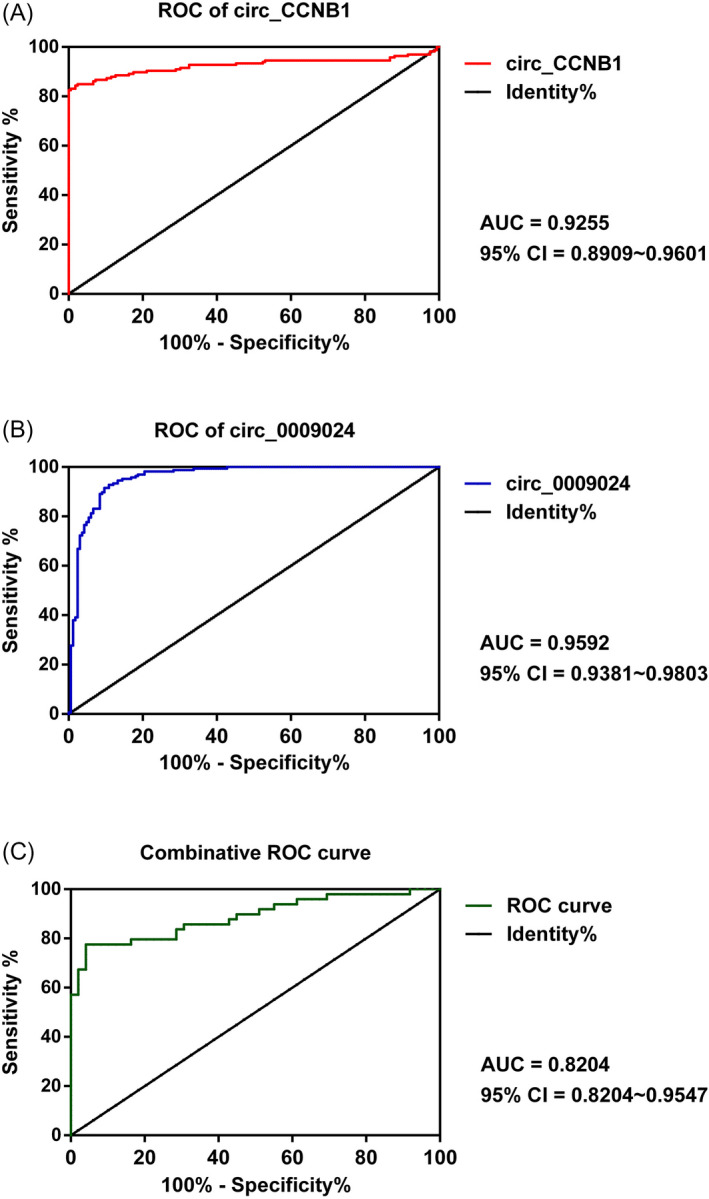
Receiver‐operating characteristic (ROC) curves for detecting T2DM from controls. A, has_circ_CCNB1. B, has_circ_0009024. C, Combinative ROC curve. ROC, receiver operating characteristic

In order to detect the diagnostic values of has_circ_CCNB1 and has_circ_0009024 for T2DM, ROC curve analysis was carried out accordingly (Figure 4). The results demonstrated the AUC of has_circ_CCNB1 and has_circ_0009024 were 0.9255 (95% CI = 0.8909‐0.9601) and 0.9592 (95% CI = 0.9381‐0.9803), respectively. Moreover, the combinative ROC curve result was 0.8875 (95% CI = 0.8204‐0.9547).

## 9 DISCUSSION

As elucidated in previous studies, a variety of circRNAs are being identified as potential noninvasive, rapid, sensitive, and specific biomarkers for different diseases providing novel prognostic or diagnostic measures. For instance, circFARSA is abundant in lung cancer cell and may function as a potential noninvasive biomarker in lung cancer.^10^ Another study proposed that circRNA Cdr1as was elevated in cholangiocarcinoma tissues serving as a potential vicious molecular biomarker to predict the worse prognosis for cholangiocarcinoma patients.^11^ Several circRNAs are reported to be involved in regulating T2DM processes, and however, their diagnostic values remain to be further verified. Moreover, a number of circRNAs participated in regulating metabolic diseases, such as osteoporosis and diabetes mellitus.^12^, ^13^ A previous study pointed out that has_circ_CCNB1 was significantly elevated while has_circ_0009024 was down‐regulated in T2DM patients,^13^ which was consistent with our results.

The triggering mechanisms of the inflammation in T2DM have been reported recently. As known, inflammatory response may trigger insulin resistance which likely contribute to T2DM occurrence and lead to complex long‐term complications.^14^ Another study reported that adipose tissues inflammation is associated with insulin resistance and serve as key factor in the development of T2DM.^15^ In our study, we examined two typical inflammatory cytokines IL‐6 and TNF‐α as indicators. IL‐6 is a multifunctional cytokines which has been found to be promising biomarker for T2DM.^16^ Moreover, elevated expression of IL‐6 was considered to be related to development of T2DM and insulin resistance.^17^ TNF‐α, an adipocytokine, which has been implicated in the development of insulin resistance, was considered to predict T2DM development as well.^18^ Another report also proposed that elevated expressions of IL‐6 and TNF‐α were associated with T2DM, functioning as potential biomarkers for T2DM.^19^ The results elucidated that has_circ_CCNB1 was positively correlated whereas has_circ_0009024 was negatively correlated with IL‐6 and TNF‐α, which contribute to confirming the accuracy of has_circ_CCNB1 and has_circ_0009024 in the diagnosis of T2DM. GLU and GHb are golden criteria for detecting diabetes mellitus from healthy controls. A previous report suggested that a threshold of GHb ≥ 43 mmol/mol in combination with an GLU ≥ 7.0 mmol/L produce a satisfactory sensitivity and specificity for diagnosis of T2DM.^20^ Hence, increased levels of GLU and GHb may identify T2DM patients from healthy controls. In the present study, the expression of has_circ_CCNB1 was positively correlated while has_circ_0009024 was negatively correlated with and GHb, indicating their potential capabilities in detecting T2MD. Moreover, the ROC analysis further demonstrated examining expressions of has_circ_CCNB1 and has_circ_0009024 is an accurate measure to distinguish T2MD.

In conclusion, the present study elucidated that has_circ_CCNB1 was increased and has_circ_0009024 was decreased in patients with T2DM. Furthermore, the expressions of has_circ_CCNB1 and has_circ_0009024 were associated with GLU, GHb and inflammatory factors, indicating these two circRNAs may be involved in pathogenesis. More importantly, the ROC analysis verified has_circ_CCNB1 and has_circ_0009024 may be novel biomarkers for detecting T2DM with high sensitivity and specificity.

## CONFLICT OF INTEREST

None.
